# The prevalence of undiagnosed chronic obstructive pulmonary disease in a primary care population with respiratory tract infections - a case finding study

**DOI:** 10.1186/1471-2296-12-122

**Published:** 2011-11-03

**Authors:** Hanna Sandelowsky, Björn Ställberg, Anna Nager, Jan Hasselström

**Affiliations:** 1 Center for Family and Community Medicine (CeFAM), Department of Neurobiology, Care Sciences and Society, Karolinska Institutet, Alfred Nobels Allé 12, S-14183 Huddinge, Sweden; 2 Bollmora Primary Health Care Center, Bollmoravägen 14, S-13540 Tyresö, Sweden; 3 Department of Public Health and Caring Sciences, Family Medicine and Clinical Epidemiology, Uppsala University, BMC, Box 564, S-75122 Uppsala, Sweden

## Abstract

**Background:**

Chronic obstructive pulmonary disease (COPD) is an underdiagnosed cause of morbidity and mortality worldwide. Prevalence of COPD has been shown to be highly associated with positive smoking history and increasing age. Spirometry is the method used for diagnosing COPD. However, identifying patients at risk of COPD to undergo spirometry tests has been challenging. Therefore, there is a need for new cost-effective and feasible diagnostic screening procedures for use in primary care centers. Our aim was to describe the prevalence and severity of undiagnosed COPD in a group of patients with respiratory infections attending urgent primary care, and to identify those variables in patients' history that could be used to detect the disease.

**Methods:**

Patients of 40-75 years (n = 138) attending urgent primary care center with acute respiratory tract infection, positive smoking history and no previously known pulmonary disease underwent pre- and post bronchodilator spirometry testing four to five weeks after the acute infection. Prevalence and severity of COPD were estimated following the Global Initiative for COPD (GOLD) criteria. Variables such as sex, age, current smoking status, smoking intensity (pack years) and type of infection diagnosis were assessed for possible associations with COPD.

**Results:**

The prevalence of previously undiagnosed COPD in our study group was 27%, of which 45% were in stage 1 (FEV1 ≥ 80% of predicted), 53% in stage 2 (50 ≤ FEV1 < 80% of predicted), 3% in stage 3 (30 ≤ FEV1 < 50% of predicted) and 0% in stage 4 (FEV1 < 30% of predicted). We found a significant association between COPD and age ≥ 55 (OR = 10.9 [95% CI 3.8-30.1]) and between COPD and smoking intensity (pack years > 20) (OR = 3.2 [95% CI 1.2-8.5]). Sex, current smoking status and type of infection diagnosis were not shown to be significantly associated with COPD.

**Conclusion:**

A middle-aged or older patient with any type of common respiratory tract infection, positive smoking history and no previously known pulmonary disease has an increased likelihood of having underlying COPD. These patients should be offered spirometry testing for diagnosis of COPD.

## Background

Chronic obstructive pulmonary disease (COPD) is an underdiagnosed cause of morbidity and mortality worldwide [1], with an estimated prevalence of 3-12% [2-4]. COPD causes suffering and adds substantial burden to national healthcare budgets [5]. An early diagnosis may motivate smoking cessation which is the only measure known to radically improve future prospects for the patient [6].

The prevalence of COPD increases considerably with age and intensity of smoking, and can vary from 25% in a general smoking population to approximately 50% in the elderly smoking population [4,7-9]. Most subjects with undiagnosed COPD have a mild form of the disease. Underdiagnosis most frequently involves patients at early stages of COPD; approximately 95% of those in stage 1 (mild COPD) and 80% of those in stage 2 (moderate COPD) remain undiagnosed [10].

Spirometry is the basis for diagnosis [11]. However, primary care providers, who often meet patients with respiratory symptoms, do not always have access, time or adequate training to use this method [12]. Alternatively, symptom-based questionnaires are available to enhance COPD screening in primary care [13,14], but are often considered time-consuming in a typical urgent care setting. To identify patients at risk of COPD and test them using spirometry has been challenging [15]. For all the reasons mentioned above, easy diagnostic screening procedures that are feasible in primary care settings are much needed [16,17]

Due to histopathological and immunological changes in their respiratory tract, smokers are more susceptible to prolonged complicated infections [18]. There is increasing evidence implicating viral infections of the respiratory tract to exacerbation of COPD. Thus, symptoms of both upper and lower respiratory tract infection may in fact be signs of underlying COPD [19-22]. There is a need for more studies into new methods for early detection of COPD. At the same time, different ways of "case finding" have been discussed [17,23]. Symptoms of an underlying chronic lung disease may become obvious when a patient develops an acute respiratory tract infection, since patients often become habituated to their symptoms and do not report them to their physicians during consultations [24]. In light of this, spirometry testing on smokers with respiratory tract infections could constitute a targeted approach to screening for underlying COPD and was assessed here for the first time.

We conducted a cross-sectional study whose aim was to measure the prevalence and severity of undiagnosed COPD among urgent care patients with respiratory tract infections who had a positive smoking history but no prior pulmonary disease diagnosis. An additional aim for this study was to explore whether simple variables in a patient's history coupled with the respiratory infection could be indicative of COPD.

## Methods

**Figure 1:**
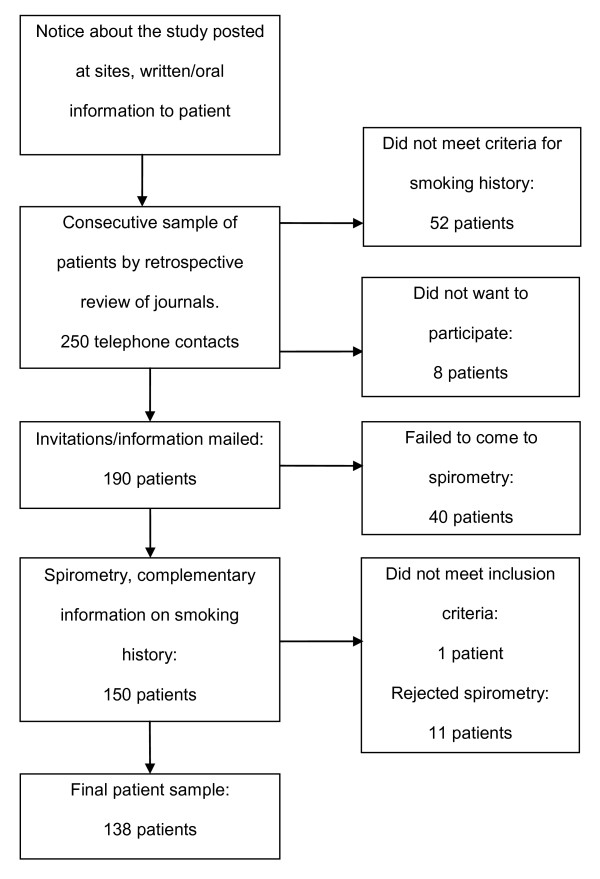
**Study enrolment summary**.

Patients were invited to participate in the study between January-March 2005 when they sought medical attention due to respiratory tract infections. All patients had visited either a primary health care center or an urgent primary care unit in a suburban area of Stockholm, Sweden. Patients, aged 40-75 years, who were eligible and agreed to participate were either current smokers (or smoking-free for no more than 6 months), or ex-smokers who had come to an urgent primary care unit because they had experienced symptoms of respiratory infection and had received subsequent diagnosis (Table 1). Patients with poor knowledge of Swedish, severe cardiac, psychiatric or multi-organ disease, prior history of lung disease (except for asthma) and those on medication with beta-blockers were excluded. A consecutive sample of patients with an ICD-10 diagnosis for respiratory tract infections was then extracted from the medical records. The diagnoses were subsequently validated by reviewing the medical records. Patients who fit the inclusion criteria were contacted by telephone. Telephone conversations were complemented with formal written invitations sent by post together with information about the study. According to the enrolment plan (Figure 1), which had been approved by the Regional Board of Ethics in Stockholm, Sweden, informed verbal consent was obtained both by telephone and prior to spirometry testing. Spirometry was performed four to five weeks after patients were diagnosed with acute respiratory infection, when respiratory function was no longer considered to be affected by the infection [25]. Participants were also asked to provide information about their smoking intensity ("pack years", table 2). An "office desk" spirometer (Vitalograph Alpha spirometry) that was calibrated daily according to manufacturer's instructions was used. A reference equation by the European Community for Coal and Steel (ECCS) was chosen for reference values [26]. All patients showing airway limitations (FEV1/FVC < 0.7) on the baseline test were given a beta-2 agonist - 8 micrograms of formoterol via inhaler (Oxis^® ^Turbuhaler^®^). Post-bronchodilator spirometry was performed after 15 minutes. The COPD diagnosis was made using the Global Initiative for Chronic Obstructive Lung Disease (GOLD) criteria [11]. Patients who needed further medical attention were referred to their family physicians.

**Table 1:** Infection diagnoses according to ICD-10

Upper respiratory tract infection	J00-J06	Acute infections in the upper respiratory system (nasopharyngitis, sinusitis, pharyngitis, tonsillitis, laryngitis, obstructive laryngitis, acute upper respiratory infection NOS)
Lower respiratory tract infection	J11-J18	Pneumonia

	J20-J22	Other acute lower respiratory infections (bronchitis, bronchiolitis, acute lower respiratory infection NOS)

	R05	Cough*

Viral infection or influenza	B34	Viral infection, unspecified

	J10	Influenza

* Patients having predominantly symptoms of lower respiratory tract infection but receiving diagnosis code R05.

**Table 2:** Description of 138 patients with respiratory tract infections seeking urgent primary care

Variables	Total	COPD		Non-COPD
**N **(%)	**138**	38 (27)		100 (73)

**Age**				

Years, mean [95%CI]	55 [54-57]	62 [59-64]		53 [51-55]
Agegroup 40-54 years n (%)	62	5 (13)		57 (57)
Agegroup 55-70 years n (%)	76	33 (87)		43 (43)

**Smoking intensity**				

Pack years*, mean [95%CI]	24 [22-26]	32 [27-36]		21 [19-24]
Pack years*, median [range]	20 [5-75]	31 [5-75]		20 [5-56]

**Smoking status**				

Current smokers, n (%)	73	25 (65.8)		48 (48.0)
Former smokers, n (%)	65	13 (34.2)		52 (52.0)

**Diagnosis**				

Upper resp. infection, n (%)	57	15 (39.5)		42 (42.0)
Lower resp. infection, n (%)	79	23 (60.5)		56 (56.0)
Viral infection/influenza, n (%)	2	0 (0)		2 (2.0)

**Sex**				

Female, n (%)		19 (50)		58 (58)
Male		19 (50)		42 (42)

**Spirometry**				

FEV1% of predicted, mean [95%CI]	95.7 [92.6-98.8]	77.5 [71.2-84.0]		102.6 [100.2-105.1]
FEV1/FVC ratio, mean [95%CI]	0.73 [0.71-0.75]	0.60 [0.57-0.63]		0.78 [0.77-0.79]

COPD severity**		**N**	**%**	

Stage 1 (mild, FEV1 ≥ 80% of predicted)		17	(44.7)	

Stage 2 (moderate, 50% ≤ FEV1 < 80% of predicted)		20	(52.6)	

Stage 3 (severe, 30% ≤ FEV1 < 50% of predicted)		1	(2.6)	

Stage 4 (very severe, FEV1 < 30% of predicted)		0	(0)	

Numbers in total and divided into groups with or without COPD.

*Number of pack years = (number of cigarettes smoked per day × number of years smoked/20)

** According to Global Initiative for Chronic Obstructive Lung Disease (GOLD)

### Statistical analysis

The statistical analysis was performed using STATA, version 8 [27]. Summary statistics such as means, proportions and measures of dispersion were computed using standard parametric methods. Multiple logistic regression was used to analyze variables associated with a COPD diagnosis, which also provided odds ratios and their 95% confidence intervals (CI). P-values < 0.05 were indicative of statistical significance. We chose the receiver/response operating characteristic (ROC) curve to select the most optimal prediction method, or model, for variables associated with COPD, and to discard suboptimal ones independently from class distribution. In order to show how well the model discriminated data we used standardized values describing the area under the ROC-curve (excellent = 0.90-1, good = 0.80-.0.90, fair = 0.70-0.80, poor = 0.60-0.70 and fail = 0.50-0.60). When plotting the curve, the closer the ROC curve is to the upper left corner, the higher the overall accuracy of the test [28]. Confidence intervals according to classification table were based on Wilson score method. P-values < 0.05 were indicative of statistical significance.

In order to find a reasonable sample size, we chose the prevalence of 30%, based on previous studies that resembled ours in design [9,13]. A random sample of 140 patients would have given a 95% confidence interval of ± 7% which was estimated to give a reasonable spread to meet the aims of the study.

### Ethical approval

The project was approved by the Regional Board of Ethics in Stockholm, Sweden.

## Results

Of the 138 eligible patients in urgent primary care, 38 were diagnosed with COPD, which corresponds to a prevalence of 27% (95% CI ± 7%). Prevalence among patients aged 55-75 years was 43%, whereas 8% was estimated for patients in the younger age group (p < 0.001) (table 2). There were as many men as women diagnosed with COPD in the study group, though women were significantly younger than men (mean age 59 versus 64 years), (p = 0.023). Two thirds of all COPD cases were current smokers, the majority of them women (p = 0.087). Thirty-four per cent of current smokers and 20% of ex-smokers had COPD (p = 0.061). The average number of pack years among patients with COPD was 32. There was no significant difference in the distribution of upper respiratory tract infection (URTI) and lower respiratory tract infection (LRTI) between the COPD and non-COPD groups, while COPD was nearly equally distributed in URTI and LRTI groups. Specifically, 26% of patients with URTI also had COPD, of which 47% were in stage 1 and 53% in stage 2 (none in stage 3). Similarly, 29% of patients with LRTI also had COPD, of which 44% were in stage 1, 52% in stage 2 and 4% in stage 3.

**Figure 2:**
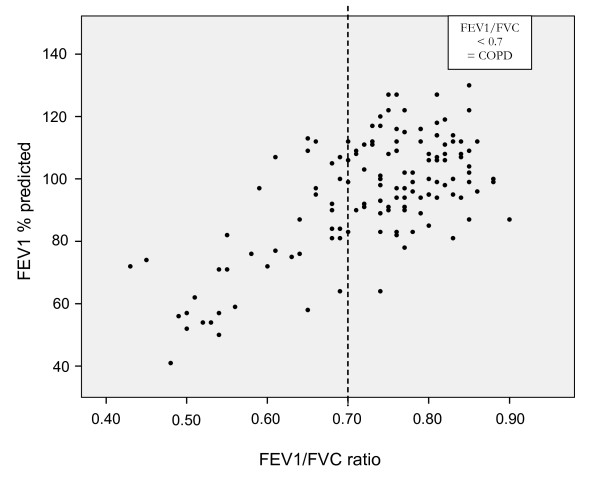
**Results of spirometry in 138 patients seeking urgent primary care for respiratory infections**.

**Figure 3:**
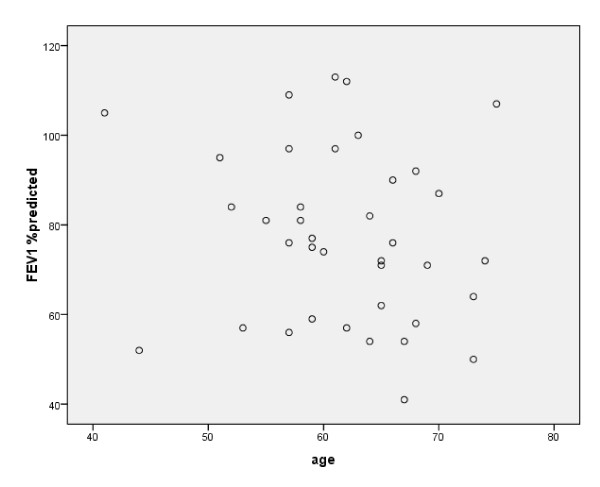
**Correlation of FEV1 (% predicted) and age (years) for all participants with COPD**.

Figure 2 shows the spirometry results of all 138 patients, plotted as FEV1 in relation to FEV1/FVC. For COPD patients, the mean FEV1/FVC was 0.60 (min 0.43, max 0.69) and the mean FEV1 was 77.5% (min 41%, max 113%) of the predicted value. Spirometry results, including severity grades of COPD and a summary of the data are shown in table 2. Lung function values were evenly distributed across all ages (Figure 3).

**Figure 4:**
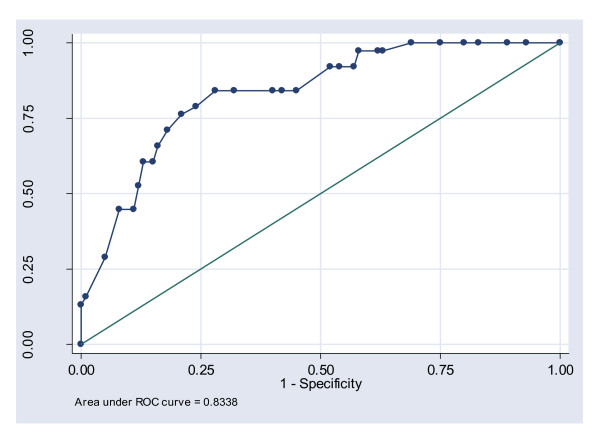
**Receiver/response operating characteristic (ROC) curve adjusted for age, pack years, current smoking, sex and infection diagnosis**.

Multiple logistic regression analysis, using COPD as a response variable, revealed a statistically significant association between COPD and age ≥ 55 years (OR = 10.9 [95% CI 3.8-30.1], p < 0.001) and between COPD and pack years ≥ 20 (OR = 3.2 [95% CI 1.2-8.5], p = 0.016), after adjustments for all explanatory variables were made (Table 3). Neither current smoking (OR = 2.5 [95% CI 0.9-6.6], p = 0.071) nor sex or location of infection diagnosis (URTI or LRTI) were significantly associated with COPD. Based on the observations above, a model for predicting COPD was created. The model included positive variables "age ≥ 55", "pack years ≥ 20" and "current smoker as positive predictors for COPD and showed a sensitivity and a specificity of 45% [95% CI 30-60%], and of 89% [95% CI 81-94%], respectively. The positive and negative predictive values were 61% [95% CI 42-76%], and 81% [95% CI 73-87%], respectively. Although variable "current smoker" did not show a significant association with COPD, it was used as a confounder since it caused the odds ratios of the top variables (age, pack years) to vary by more than 10%. The area under the receiver/response operating characteristic (ROC) curve was 0.83 (Figure 4). Finally, the proportion of correctly classified observations (COPD or non-COPD) was 77%.

**Table 3:** The odds ratio (OR) for having COPD

			COPD
**Age**	Years	≥ 55	10.9 [3.8-30.1]
		< 55	1.0

**Smoking intensity**	Pack years	≥ 20	3.2 [1.2-8.5]
		< 20	1.0

**Smoking status**	Current smoker	yes	2.5 [0.9-6.6]
		no	1.0

**Diagnosis**		Upper respiratory infection	0.8 [0.3-2.0]
		Lower respiratory infection	1.0

**Sex**		female	0.9 [0.3-2.1]
		male	1.0

Odds ratios adjusted for pack years, age, current smoking status, diagnosis and sex with 95% confidence intervals.

Nineteen percent (n = 40) of patients who were invited to undergo spirometry failed to show for their appointments. The dropout rate was independent of sex, age and infection diagnosis. However, smoking status (current or ex-smoker) and intensity (pack years) of patients who dropped out were not recorded.

## Discussion

There is an ongoing discussion about how to improve early detection of COPD. In this case finding study we found a high prevalence (27%) of previously undiagnosed COPD. Statistical analysis showed that assessing three simple variables (age, smoking intensity and smoking status) among patients with any kinds of acute respiratory infections attending urgent primary care could be a feasible and effective way to short-list those who need spirometry testing to confirm the presence of underlying COPD. Moreover, preliminary screening for airflow obstruction using a handheld spirometer prior to full spirometry testing may be an efficient way of finding new COPD cases [29].

In comparison with other case finding methods, such as questionnaires and random screenings of smokers this study utilized a targeted approach in a real-life context [9,13,23,30]. In addition, such an approach would be suitable in urgent care settings, where consultation time is usually limited. Questionnaires may be more appropriate in clinical settings other than urgent primary care where more time for consultation is available.

The distribution patterns of different stages of COPD matched previous prevalence estimates [7,9,10] and indicated early COPD. The distribution of stage 1 was found to be independent of age ruling out the possibility of overdiagnosis among the elderly persons in this study population. Instead, 53% of undiagnosed COPD cases were in stage 2 (moderate) and 45% in stage 1 (mild) suggesting not only that underdiagnosis of COPD may be common but also that underdiagnosis of moderately advanced stages of COPD may be common. In effect, underdiagnosis has been shown in earlier epidemiological studies of COPD among primary care patients [9,11,31]. These studies have shown that underdiagnosis of COPD occured partly because of "doctor's delay", i.e., doctors who don't suspect underlying COPD [32,33] and partly because of "patient's delay", i.e, patients who self-report good health [34].

The major strength of the study is its connection and relevance to real-life contexts in primary health care. Our case finding approach suggests that both patients and doctors play an active role in the early detection of COPD which, in turn, may lead to new diagnoses and patients with increased motivation to quit smoking [35].

To our knowledge, no case finding study has been performed in a study group similar to ours. A high prevalence of COPD among patients with LRTI and positive smoking history has been shown earlier [31]. Interestingly, a fourth of patients with URTI in our study had a previously undiagnosed COPD and prevalence among all patients was independent of the infection site, i.e, upper or lower respiratory tract. However, due to the small sample size, conclusions should be drawn with caution. More studies on the role of the infection site in the detection of COPD are needed. Prevalence of COPD in our study population was consistent with that observed in earlier studies [9,11,31]. Prevalence of undiagnosed COPD among smokers is largely known to be approximately 20-50% depending on smoker's age and smoking intensity [8].

Although the women with undiagnosed COPD in the group were younger than the men, we found no association between prevalence of COPD and sex. This finding was inconsistent with earlier studies, though the small sample size may explain the discrepancy. Recent evidence has suggested that women are at higher risk of developing COPD than men accompanied by an earlier onset and a more severe clinical manifestation of the disease [36,37]. Prevalence of COPD among women is likely to increase due to changes in smoking habits of women [38].

The positive (61%) and negative (81%) predictive values of our model, which utilized three variables (age, smoking status and intensity) to detect COPD in patients with an acute respiratory tract infection were relatively high. A possible explanation for this outcome may be that multiple regression analysis was influenced by the presence of random factors typical for small-sample studies.

Having said that, the small sample size was the main limitation of this study. In addition, the exclusion of patients aged 75 years or over and patients with severe cardiac disease and severe psychiatric diagnoses, among whom prevalence of COPD has been shown to be high [39], is another notable limitation of this study. The reason for excluding those patient groups was the particular focus of this study on patients who do not normally attend primary care. As a consequence, we may have observed a lower prevalence of COPD than that reported in studies with broader inclusion criteria [14,40]. Also, although we were aware that the optimum time for performing spirometry on patients with COPD was six to seven weeks after an exacerbation [41], we chose to perform the test on our study population four to five weeks after infection onset since we had no prior knowledge of who had underlying COPD.

## Conclusion

COPD is a potentially life-threatening disease whose progress can be slowed relatively easily and inexpensively by smoking cessation. We conclude that patients aged 40 or over with a positive smoking history (more than 20 pack years) who develop any type of respiratory infection may also have underlying COPD. Thus, it is crucial for physicians to identify this high-risk group and offer them spirometry testing for early detection of COPD, which may motivate those patients to quit smoking.

## Competing interests

The authors declare that they have no competing interests and are solely responsible for the content of the paper.

## Authors' contributions

HS participated in the design of the study, data collection and organization, performing spirometry testing, statistical analysis and manuscript drafting and critical revising. BS advised on and participated in statistical analyses, manuscript drafting and critical revising. AN participated in manuscript drafting and critical revising. JH participated in the design of the study and in manuscript drafting and critical revising. All authors read and approved the final version of the manuscript.

## Pre-publication history

The pre-publication history for this paper can be accessed here:

http://www.biomedcentral.com/1471-2296/12/122/prepub
